# THE POTENTIAL OF EXTENDED REALITY SOLUTIONS TO SUPPORT OLDER ADULTS WITH AND WITHOUT COGNITIVE IMPAIRMENTS

**DOI:** 10.1093/geroni/igae098.1185

**Published:** 2024-12-31

**Authors:** Walter Boot, Andrew Dilanchian, Saleh Kalantari

**Affiliations:** Weill Cornell Medicine, New York, New York, United States; Florida State University, Tallahassee, Florida, United States; Cornell University, Ithaca, New York, United States

## Abstract

We are living in an era of rapid technological innovation, which presents novel opportunities for technology to foster and enhance the independence, productivity, health, safety, social connectivity, and quality of life of older people with and without cognitive impairments. Both established and emerging technologies can play significant roles in achieving these aims, but to meet their fullest potential they must be designed with a user-centered approach that considers the needs, abilities, and preferences of diverse populations of older people, should be evaluated for usability, and rigorously assessed to determine if they are achieving their intended outcomes. This talk will focus specifically on the potential of novel Extended Reality (XR) solutions, including Virtual Reality (VR) and Augmented Reality (XR), to support activities vital to older people’s brain health, wellbeing, and independence. It will begin with an introduction to the capabilities of these technologies and how these technologies are well-suited to support aging adults, followed by the potential of these tools to support older people with and without cognitive impairments in domains such as cognitive health, social connectivity, wellness, way-finding, and leisure. Evidence for the efficacy of these interventions will be summarized, followed by a presentation of best practices and design guidelines for the use of XR among aging persons based on the latest empirical evidence. We will conclude with a discussion of the future of these technologies and outline a research agenda moving forward.

